# Neurovascular crosstalk and cerebrovascular alterations: an underestimated therapeutic target in autism spectrum disorders

**DOI:** 10.3389/fncel.2023.1226580

**Published:** 2023-08-24

**Authors:** Yiran Wang, Shunyu Yu, Mengqian Li

**Affiliations:** ^1^Queen Mary School, Jiangxi Medical College, Nanchang University, Nanchang, China; ^2^Department of Psychosomatic Medicine, The First Affiliated Hospital of Nanchang University, Nanchang, Jiangxi, China

**Keywords:** autism spectrum disorder (ASD), cerebrovascular, blood-brain barrier, neurovascular unit, neurovascular crosstalk

## Abstract

Normal brain development, function, and aging critically depend on unique characteristics of the cerebrovascular system. Growing evidence indicated that cerebrovascular defects can have irreversible effects on the brain, and these defects have been implicated in various neurological disorders, including autism spectrum disorder (ASD). ASD is a neurodevelopmental disorder with heterogeneous clinical manifestations and anatomical changes. While extensive research has focused on the neural abnormalities underlying ASD, the role of brain vasculature in this disorder remains poorly understood. Indeed, the significance of cerebrovascular contributions to ASD has been consistently underestimated. In this work, we discuss the neurovascular crosstalk during embryonic development and highlight recent findings on cerebrovascular alterations in individuals with ASD. We also discuss the potential of vascular-based therapy for ASD. Collectively, these investigations demonstrate that ASD can be considered a neurovascular disease.

## 1. Introduction

Autism spectrum disorder (ASD), also known as autism, is a heterogeneous neurodevelopmental disorder characterized by deficits in social interaction and communication, accompanied by repetitive behaviors (Schafer et al., 2019). In this review, we will use the term “autism” to refer to ASD in general for brevity. In 2020, the estimated prevalence of ASD among 8-year-old children in America was approximately one in 36 (Maenner et al., 2023). ASD is typically diagnosed in early childhood and is considered a lifelong condition (Lord et al., 2018). Despite the high prevalence and substantial impact of ASD, the development of effective therapies is hindered by our limited understanding of its underlying pathogenesis. Behavioral interventions are the primary approach for managing ASD. Although antipsychotics such as risperidone and aripiprazole are used for ASD-related irritability, there are no approved medications specifically targeting the core symptoms of autism, such as social communication deficits and repetitive behaviors (Neul and Sahin, 2015; Xu et al., 2019). The lifespan cost of supporting a person with ASD in the United States was about $2.4 million, and by 2025, the financial burden of ASD may far exceed the costs of diabetes and attention deficit hyperactivity disorder (Buescher et al., 2014; Leigh and Du, 2015). ASD places a significant burden on families, society, and the economy, suggesting that further research into the pathogenesis of ASD and finding a safe, effective, reliable, and inexpensive treatment strategy is urgent (Buescher et al., 2014). According to many epidemiological reports, ASD is caused by a complex interplay of genetic susceptibility and/or environmental factors, and potentially immunological influences (Theoharides, 2013; Colvert et al., 2015; Geschwind and State, 2015; Modabbernia et al., 2017). Current research on the etiology of ASD has mainly focused on identifying risk genes and neurological abnormalities (Hazlett et al., 2017; Satterstrom et al., 2020). Neuropathological investigations have revealed abnormal brain development during the early prenatal period in individuals with ASD (Rodier, 2002), involving disrupted functional connectivity (Courchesne and Pierce, 2005), cortical hyperexpansion (Hazlett et al., 2017), altered neuronal plasticity (Ardalan et al., 2022), and extensive atypical synaptic function (Johnson et al., 2015). However, the precise pathogenesis of ASD remains elusive.

The brain accounts for at least 20% of the body’s resting metabolic energy consumption, primarily dedicated to supporting neuronal activity (Magistretti and Allaman, 2015). The brain vasculature develops into a unique and efficient vascular system different from the peripheral vascular system, to maintain enormous energy consumption and normal brain function. Recent insights have expanded our understanding of the neurovascular interactions beyond traditional oxygen and nutrient supply. Cerebrovascular elements also emerge as critical scaffolds and paracrine signaling mediators, regulating the development, function, and maintenance of various neural cell populations (Paredes et al., 2018; Segarra et al., 2019). The precise interaction between the nervous and cerebrovascular systems is essential for normal development, functional maintenance, and aging of the central nervous system (CNS) (Peguera et al., 2021). In recent years, there has been growing interest in exploring the role of cerebrovascular abnormalities in neurodevelopmental and neurodegenerative diseases, such as schizophrenia and Alzheimer’s disease (Nation et al., 2019; Ouellette and Lacoste, 2021). The potential contribution of cerebrovascular abnormalities to ASD has only recently been gradually described (Ouellette et al., 2020). Although some studies have reported the presence of cerebrovascular abnormalities in individuals with ASD, its contribution to the pathophysiology of ASD remains incompletely understood. In this work, starting from the process of cerebrovascular formation during embryonic development, we review research findings on cerebrovascular alteration in ASD patients. By exploring the association of specific cerebrovascular characteristic defects with neurological abnormalities and behavioral disturbances in ASD, this review aims to enhance our understanding of ASD’s pathogenesis and provide a theoretical foundation for the development of novel therapeutic interventions.

## 2. Neurovascular development in the central nervous system

Neurovascular development refers to the parallel emergence and formation of the neural and cerebrovascular during early embryonic stages (Bautch and James, 2009). While the vascular and nervous systems each establish distinct specialized networks, they also share overlapping mechanisms. This integration of signaling pathways and cellular responses is crucial for normal brain development, function, and aging (Ouellette and Lacoste, 2021). In mice, between embryonic day 8.5 (E8.5) and 10 (E10), the dorsal ectoderm region containing neuroectodermal cells undergoes specialization into the neural plate. Subsequently, the neural plate elongates, folds, and closes, forming the neural tube (Nikolopoulou et al., 2017). Incipient neural tubes have no vessels, this mild “physiological hypoxic” environment promotes the proliferation and differentiation of neural stem cells (NSCs). This process is likely mediated through the hypoxia-inducible factor-1 (HIF-1) signal pathway (Zhu et al., 2005). Studies involving conditional knockdown of HIF1-α in mouse midbrain-derived neural precursor cells (mNPCs) have demonstrated an impaired midbrain-specific proliferation of mNPCs and a significant reduction in dopaminergic differentiation *in vitro*. Notably, this phenotype could be rescued by 50 ng/ml VEGF treatment (Milosevic et al., 2007). Conversely, the increasing oxygen and nutrient demands of immature neurons serve as signals to promote angiogenesis (Ouellette and Lacoste, 2021). Simultaneously to the closure of neural tube, the angioblasts (endothelial cell precursors) in the adjacent presomitic mesoderm are recruited by neural tube to form perineural vascular plexus (PNVP) which constitutes the initial vascular network in CNS (James and Mukouyama, 2011). Vascular endothelial growth factor A (VEGF-A), a downstream signal of the transcription factor HIF-1 primarily secreted by the CNS-resident NPCs, acts as the initial driver to trigger angiogenesis (Ferrara et al., 2003; James et al., 2009; Peguera et al., 2021). Thereafter CNS vascularization accompanies its development, followed by massive neurovascular molecular communications. VEGF-A facilitates tip cell migration and enhances stalk cell proliferation at the tips of vascular sprouts, promoting vascular growth into non-vascular regions (Gerhardt et al., 2003). Around E10.5, vessel sprouts from the PNVP invade the neuroectoderm, extending from the pial surface to the luminal surface, resulting in the formation of the intraneural vascular plexus (INVP) (James and Mukouyama, 2011). In general, numerous angiogenic sprouts originating from the IVNP follow the radial glia cells (RGCs) fibers, branch laterally, and anastomose to form the periventricular vascular plexus (PVP) within the ventricular zone (VZ) (Tata and Ruhrberg, 2018). Subsequently, the periventricular and perineural vessels coalesce, forming a complex network of blood vessels intertwined with neural cells that undergo dynamic remodeling until postnatal stage. The instructive role of the vascular system in CNS fate has been largely overlooked in the past but is now garnering increasing attention.

At the same time as neural tube vascularization begins, RGCs transformed from NSCs, present a bipolar morphology, with a long in basal side vessels in the meningeal surface (Sun and Hevner, 2014). RGCs are a major class of neural progenitor cells as well as serve as scaffolds for pyramidal neuron migration in cortical formation (Sun and Hevner, 2014). A 2018 study identified the significant role of CNS vascular endothelial cells (ECs) in regulating neuronal migration and differentiation (Segarra et al., 2018). Reelin, a large glycoprotein secreted by Cajal-Retzius cells, plays a crucial role in neocortex development. Knockdown of reelin/Dab1 signaling specifically in the vascular system impaired the anchoring of RGCs fibers on pial vessels, induced altered pyramidal neuron location, and affect the differentiation of glial cells to neuronal cells (Segarra et al., 2018). This suggests that the vasculature may direct neuronal migration through the function of Dab1 on ECs. Moreover, reelin interacts with the VEGF/VEGF2 pathway to control EC proliferation and vascular filopodia expansion, exerting a powerful proangiogenic effect (Segarra et al., 2018). As a good example, indicated that Reelin/Dab1 signals perform bivalent functions to coordinate neurovascular communication. In addition, vascular ECs around the stem cell niche can promote NPCs proliferation by secreting Neuropilin 1 (NRP1), as one way of vascular regulation of neurogenesis (Tata et al., 2016). The neuropilin (NRP) family members NRP1 and NRP2 also work as receptors or co-receptors of VEGF and semaphorins to modulates angiogenesis (Simons et al., 2016). Knockdown of endothelial uncoupling protein 2 (UCP2) in ECs inhibited NPCs differentiation into neuronal but promoted differentiation into astrocyte, this suggests that blood vessels near NPCs influence neurogenic-to-gliogenic transition (Wang et al., 2022). Conversely, Wnt signaling is crucial for guiding cerebral angiogenesis during neural development. NPCs expressing Wnt7a/b ligand stimulate sprouting angiogenesis of the INVP and PNVP, promoting invasion of the neural tube and inducing and maintaining blood-brain barrier (BBB) properties (Stenman et al., 2008; Daneman et al., 2009). This process involves the interaction of Gpr124 (an orphan GPCR) expressed by ECs with the Norrin/Frizzled4 signaling pathway (Zhou and Nathans, 2014). Throughout the embryonic and postnatal stages, NPCs undergo a highly active process of cell division, differentiation, and migration to establish the intricate and organized CNS. Overall, balanced neurovascular interactions are essential to support the development of the CNS and cerebrovascular systems.

During vertebrate development, specialized blood vessels are formed to meet the specific requirements of different tissues. The formation of the BBB coincides with the growth of vasculature into the CNS. BBB is a specialized vascular barrier between circulating blood and nervous system, acting as a gatekeeper to maintain homeostasis (Langen et al., 2019). It consists of a specialized monolayer of brain capillary ECs. These cells exhibit unique cytological features that differ from those of peripheral ECs, including specialized tight junctions (TJs) between cells, designated transporters that highly selectively control specific substrates and low rates of transcellular vesicle trafficking (Langen et al., 2019). However, early transplantation experiments demonstrated that the BBB properties are induced by the perivascular cellular (pericytes and astrocytes) and CNS environment, rather than the intrinsic properties of CNS endothelial cells (Stewart and Wiley, 1981; Daneman et al., 2010). During embryonic development, the BBB gradually forms from the vascularization of the CNS. At an early stage of cerebrovascular development (sprouts from PNVP), they already acquire TJs under the regulation of canonical Wnt signaling (Risau, 1991) and express barrier function-related genes such as CLDN-5 (Morita et al., 1999). However, blood vessels do not exhibit barrier properties in the early stages of development due to the continued active transcellular transport of ECs. The inhibition of EC endocytosis gradually occurs as the barrier forms (Langen et al., 2019).

**FIGURE 1:**
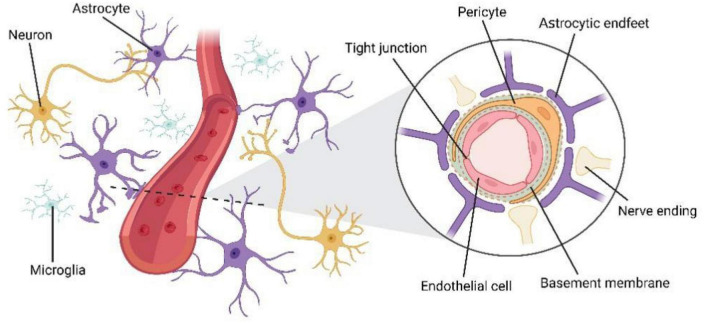
The structure of neurovascular unit (NVU). Endothelial cells are connected by tight junctions, surrounded by pericytes, and embedded in the basement membrane, the end-foot processes of astrocytes surround blood vessels, forming NVU.

Previous physiological studies on cerebral blood flow (CBF) dynamics have demonstrated intensive intercellular communication between cells of the vasculature and nearby neurons and glial cells (McConnell et al., 2017). The BBB is now understood to function as part of a multicellular unit called the neurovascular unit (NVU) rather than operating independently (Figure 1) (McConnell et al., 2017). Endothelial cells are connected by tight junctions, surrounded by pericytes, and embedded in the basement membrane, the end-foot processes of astrocytes surround blood vessels, forming NVU (Abbott et al., 2006; Tam and Watts, 2010). Compared with peripheral blood vessels, cerebral vessels require dense vascularization to maintain the supply of nutrients and oxygen and precise control of CBF (Ouellette and Lacoste, 2021). In the mammalian brain, cerebral vessels respond to the needs of the nervous system by increasing the rate of CBF and oxygen transport, a mechanism known as neurovascular coupling (Iadecola, 2017). The integrity of the NVU is critical for this normal function (Stobart and Anderson, 2013). Besides, the NVU induces and regulates BBB establish (Abbott et al., 2006), and controls the selective transportation of substances in the CNS (Attwell et al., 2010; Daneman and Prat, 2015; Iadecola, 2017). Therefore, the interaction among the cellular elements of the NVU is crucial for maintaining CNS homeostasis (Segarra et al., 2019).

The neuronal activity also ensures the development of the cerebrovascular network. In the stress model of rats with social isolation, occurs BBB damage early, manifested as increased BBB permeability accompanied by increased IL-6 expression (Schiavone et al., 2017). Chronic stimulation of neonatal but not adult mice, such as repetitive sounds, whisker deflection, and chemically induced seizures, reduced endothelial proliferation and vascular sprouting, which may be attributed to inhibition of vascular growth by abnormally active interneurons and glial cells through the release of nitric oxide (Whiteus et al., 2014). Exosomes provide another mechanism for mediating neurovascular communication in the CNS. Exosomes are extracellular vesicles enriched in proteins and lipids that are produced by nearly every cell type and play a significant role in neuron-glial cell communication (Frühbeis et al., 2012; Tkach and Théry, 2016). These exosomes carry microRNAs, which can cross the BBB and regulate gene expression in various physiological and pathological processes of the CNS through the RNA-induced silencing complex (Tkach and Théry, 2016). In zebrafish larvae, embryonic neurons express a microRNA called miR-132 in exosomes, which regulates BBB integrity by controlling the expression of endothelial VE-cadherin. When miR-132 is antagonized, zebrafish larvae exhibit severe intracranial hemorrhage and impaired BBB integrity. This suggests exosome-mediated long-range communication between neurons and brain ECs (Xu et al., 2017). Overall, the neural and cerebrovascular systems develop and mature simultaneously. Molecular pathways shared by both systems converge during CNS development to regulate the complex processes of morphogenesis and functional formation in the brain.

## 3. Cerebrovascular deficits in autism spectrum disorders

**FIGURE 2:**
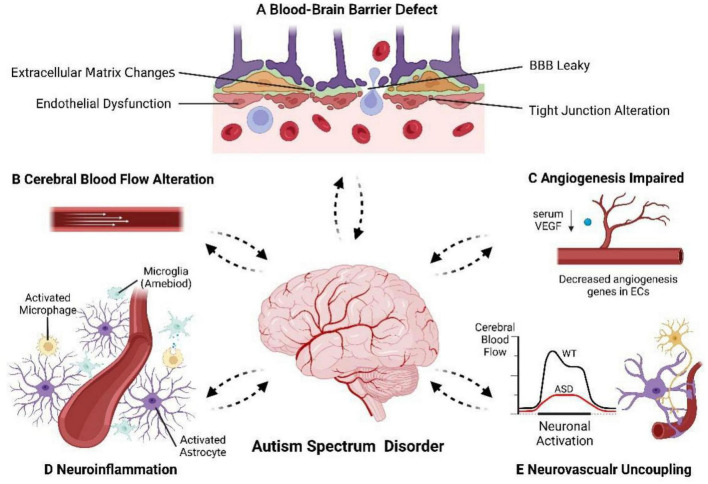
The cerebrovascular alteration in autism spectrum disorder. The cerebrovascular, especially BBB and NVU, appears to have deficits in ASD patients where we see: **(A)** BBB defect. **(B)** CBF alteration in specific brain regions. **(C)** Angiogenesis impaired. **(D)** Neuroinflammation. **(E)** Neurovascular uncoupling and decreased hemodynamic response. Dotted arrows indicated that the causal relationship between cerebrovascular abnormalities and the pathogenesis of ASD is unclear.

Prior research has suggested that the etiology of ASD might involve a complex interaction between genetic and environmental factors (Geschwind and State, 2015; Modabbernia et al., 2017). However, the molecular and cellular mechanisms underlying ASD pathogenesis remain poorly understood. While most studies have focused on neuronal aspects, ASD is now thought to be associated with abnormal vasculature. The healthy development and functioning of the brain rely heavily on a well-functioning vascular system. Given the extensive crosstalk between the vasculature and nervous system during embryonic development, it is reasonable to hypothesize that structural and functional abnormalities in the cerebral vasculature could have detrimental effects on neurodevelopment. Surprisingly, few studies have explored the neurological consequences of vascular abnormalities during the embryonic period in the context of ASD. Furthermore, the brain cannot store amounts of energy and therefore depends on a constant supply of oxygen and nutrients of cerebral vasculature, to buffer its high but variable metabolic demand. The unique characteristics of the cerebral vasculature ensure a stable microenvironment for neural activity and provide adequate oxygen and nutrients. These characteristics distinguish it from the peripheral vasculature including a dense vasculature to maintain adequate perfusion, a functional BBB to maintain brain homeostasis, as well as regulating stable local CBF in response to neuronal demand (Ouellette and Lacoste, 2021). Defects in the brain vascular system can have severe and irreversible impacts on the CNS. Increasing evidence indicates that these critical features of the cerebral vasculature are altered in individuals with ASD (Figure 2). It is not clear whether cerebrovascular abnormalities are the cause of autism or the result of pathophysiologic changes. The association between genetic, environmental factors and cerebrovascular abnormalities in ASD needs to be further explored.

### 3.1. Impaired cerebral blood supply in autism spectrum disorders

#### 3.1.1. Insights into cerebral blood flow changes in autism spectrum disorders

Common imaging techniques such as Functional magnetic resonance imaging (fMRI), Arterial Spin Labeling (ASL), single-photon emission computed tomography (SPECT), Positron Emission Tomography/Computerized Tomography (PET/CT), and an emerging technique Diffuse correlation spectroscopy (DCS) (Lin et al., 2023) have been used to detect regional cerebral blood flow (rCBF) and can significantly enhance our understanding of the contribution of brain vasculature to ASD.

A case-control study showed that significantly higher rCBF values were prevalent in specific regions in patients with ASD, and the higher the rCBF value, the more severe the socialization deficit. This may be due to alterations in metabolism and axonal function that reduce the role of nerves in cognitive and social functioning, which provokes a compensatory response from glial cells, that results in rCBF increase (Peterson et al., 2019).

#### 3.1.2. Altered vasculogenesis and angiogenesis in autism spectrum disorders

Early in embryonic development, angioblasts are recruited by the neural tube and form the perineural vascular plexus covering the entire neural tube by vasculogenesis (Risau and Wolburg, 1990). After that, the vessel branches from pre-existing vessels establishes a more complex vascular network by angiogenesis. During this process, vascular sprouts from PNVP ingress and extend from the pial surface toward luminal part (Greenberg and Jin, 2005). Subsequently the nascent cerebral vasculature recruits mural cells and forms the extracellular matrix, which undergoes formation, stabilization, branching, pruning, and specialization to acquire features adapted to the brain (Jain, 2003). E15 exposure of rats to the angiogenesis inhibitor and teratogen thalidomide causes abnormal, leaky blood vessels throughout the brain, especially in the cortex, resulting in cortical and hippocampal region malformations, which are implicated in the pathophysiology of various neurological disorders including autism (Hallene et al., 2006). This finding suggests that normal neuronal migration and maturation are reliant on embryonic angiogenesis. Drugs or events that affect vasculogenesis may promote the development of neurological deficits. The role of neuroinflammation on angiogenesis will be mentioned later. Notably, the potent anti-inflammatory and immunomodulatory effects of thalidomide, through suppression of TNF-α and NF-κB, may also underlie this effect (Talaat et al., 2015). There is growing evidence of abnormal angiogenesis in ASD. Chromosomal domain helicase DNA-binding protein 8 (Chd8) is one of the ASD risk genes (Satterstrom et al., 2020). Chd8 haploinsufficient old mice exposed to the insecticide deltamethrin showed downregulation of the vascular endothelium-associated genes Kdr and Ptprb in the cerebral cortex (Jiménez et al., 2022). However, immunohistochemical staining did not reveal significant changes in vessel length or density in the deltamethrin-exposed mice. Moreover, *de novo* mutations in brain-specific angiogenesis inhibitor (BAI1/ADGRB1) were identified in ASD patients (Satterstrom et al., 2020; Shiu et al., 2022). VEGF is an important pro-angiogenic factor that also acts as a key molecule for neuronal survival and axonal growth (Yasuhara et al., 2004; Takahashi and Shibuya, 2005). Decreased serum VEGF concentrations and increased sVEGFR-1 levels were found in patients with severe ASD (Emanuele et al., 2010). Although it is uncertain whether peripheral concentrations respond to CNS expression levels, this result predicts a reduced rate of angiogenesis. This demonstrates the presence of an anti-angiogenic environment in ASD, which may ultimately lead to abnormal perfusion as well as impaired neuronal function (Emanuele et al., 2010). However, the limited sample size in this study poses certain limitations, and other studies have yielded differing results (Kajizuka et al., 2010). It is worth noting that 16*p*11.2^*df*⁣/ +^ mice exhibited impaired cerebral angiogenesis at postnatal day 14 as well as lower the density and branching of endothelial networks throughout the cerebral cortex (Ouellette et al., 2020). This is due to endothelial dysfunction as characterized by downregulation of angiogenic genes in brain ECs from primary 16*p*11.2^*df* +^ mice and induced-pluripotent-stem-cell-derived ECs from 16p11.2-deficient human vectors (Ouellette et al., 2020). Interestingly, in addition to reduced vasculogenesis and angiogenesis, hypervascularization was also found to be present in patients with ASD. A 2016 immunocytochemical study of postmortem brain sections from ASD revealed significantly elevated angiogenic markers associated with perivascular pericytes and angiogenic endothelial precursors throughout superior temporal cortex, fusiform cortex, pons/ midbrain and cerebellum (Azmitia et al., 2016). This study indicated that angiogenesis continues after normal development in ASD patients (Azmitia et al., 2016). This persistent microvascular rearrangement in ASD may contribute to brain neuroplasticity, helping to maintain short, localized connections but inhibiting the development of long, complex brain connections required for language and social interactions (Azmitia et al., 2016). These studies collectively suggest the presence of abnormal angiogenesis in ASD and may explain the decrease/increase in CBF in different brain regions as well as the association with various ASD behavioral deficits. CNS vasculature supporter proper neuronal migration and axon pathfinding (Peguera et al., 2021). During embryonic development, vascular defects may affect many vascular-dependent neurodevelopmental processes as well as CBF in ASD.

### 3.2. Altered blood-brain barrier in autism spectrum disorders

#### 3.2.1. Blood-brain barrier permeability markers in autism spectrum disorders

The BBB is a vital boundary of the brain which consists of brain capillary endothelial cells, and its dysfunction is associated with various neurological disorders (Sweeney et al., 2019). Animal models have provided evidence linking BBB dysfunction to ASD. Solute carrier transporter 7a5 (SLC7A5) is an amino acid transporter on the BBB, which is important for maintaining the level of branched-chain amino acids in the brain. A 2016 study found that deletion of SLC7A5 in BBB ECs in mice leads to severe neurological abnormalities (Tărlungeanu et al., 2016). Homozygous mutations in the SLC7A5 gene have also been observed in patients with ASD, who exhibited unbalanced neuronal activity, autistic behavior, and motor delay (Tărlungeanu et al., 2016). Moreover, in valproic acid-induced ASD mouse model, leakage to Evans blue dye was found in the cerebellum, indicating increased BBB permeability (Kumar and Sharma, 2016). Mounting studies suggest potential alterations in the BBB in ASD individuals. Genetic screens have shown that some autism-related genes regulate BBB function to some extent. Activation of Sonic Hedgehog (Shh) and WNT/β-catenin signaling is critical for angiogenesis, BBB formation, and neurodevelopment (Alvarez et al., 2011). Multiple gene mutations associated with this pathway have been identified as ASD risk mutations that may lead to reduced and impaired regulation of CBF, BBB impairment, leakage of toxic components and defective molecular clearance, neurological dysfunction, and possibly increased incidence of ASD (Gozal et al., 2021). Markers of BBB permeability, including autoantibodies against brain endothelial cells (ECs), S100B protein, platelet-endothelial adhesion molecule-1 (PECAM-1), and vascular cell adhesion molecule-1 (VCAM-1), have been found to be altered in individuals with ASD. A subset of children with ASD had significantly higher serum levels of autoantibodies against brain ECs compared to typically developing individuals, indicating the presence of BBB defect (Connolly et al., 1999). Elevated levels of S100B, a protein produced by brain astrocytes and considered a marker of BBB breakdown, have also been observed in ASD individuals (Marchi et al., 2004; Al-Ayadhi and Mostafa, 2012). Other studies have shown that serum PECAM-1 and VCAM-1 level are lower in ASD and PECAM-1 levels are negatively correlated with head circumference of infants at birth (Tsuchiya et al., 2007; Onore et al., 2012). These adhesion molecules mediate leukocyte infiltration and regulate BBB permeability, cluing that components of the BBB may play a role in ASD (Lee and Benveniste, 1999; Blankenberg et al., 2003).

#### 3.2.2. Altered blood-brain barrier structure in autism spectrum disorders

A small number of studies directly examined BBB properties to further demonstrate BBB dysfunction in ASD. Fiorentino et al. (2016) conducted a study of the post-mortem brain of ASD patients, indicating that the expression of gene associated with BBB integrity are altered including claudin (CLDN)-5 and -12 as well as increased neuroinflammation. Tight junction proteins CLDN-3, -5, and -12 in ECs all contribute to the BBB, in particular, CLDN-5 is now recognized as the predominant isoform (Nitta et al., 2003; Greene et al., 2019). CLDN-5 deficiency is implicated in BBB disorders and has been associated with psychiatric disorders, such as depression, schizophrenia, neurodegenerative disorders, and neuroinflammatory disorders (Menard et al., 2017; Greene et al., 2019). Interestingly, elevated CLDN-5 gene expression and protein content have been consistently observed in the cortex and cerebellum of individuals with ASD (Fiorentino et al., 2016). This may suggest a compensatory mechanism for the destruction of the BBB by neuroinflammation (Fiorentino et al., 2016). An alternative explanation is that due to endothelial trafficking or protein mutations, CLDN proteins fail to be incorporated into tight junctions but continue to be produced compensatively (Fiorentino et al., 2016). Moreover, the CLDN associated with pore formation is increased in the intestine of ASD patients, but the expression of tight junction components of barrier formation is decreased, confirming the possibility of gastrointestinal barrier dysfunction in ASD (Fiorentino et al., 2016). Also in this study, matrix metalloproteinase (MMP)-9 gene expression was increased in ASD subjects and its secretion induced BBB destruction, aligning with the hypothesis of BBB damage (Fiorentino et al., 2016; Vafadari et al., 2016). Overall, this study shows directly evidences of structural alterations in the BBB in the ASD population.

#### 3.2.3. Pathological conditions of autism spectrum disorders associated with blood-brain barrier dysfunction

Although BBB impairment in individuals with ASD has been demonstrated, further research is needed to investigate the mechanisms and the extent of BBB disruption in ASD. Immune dysfunction has also been found in some patients with ASD, characterized by microglia activation and increased cytokines, as well as high expression of immune/inflammatory genes (Vargas et al., 2005; Voineagu et al., 2011; Onore et al., 2012; Gesundheit et al., 2013; Pape et al., 2019). Studies have shown significant upregulation of TSPO gene expression in the brains of ASD individuals, indicating increased microglia activation and reactive astrocyte expression, which are molecular markers of brain inflammation (Lavisse et al., 2012; Fiorentino et al., 2016). Consistent with this, individuals with ASD exhibit decreased levels of brain-derived neurotrophic factor (BDNF) and increased levels of inflammatory biomarkers and acute phase proteins in serum and/or cerebrospinal fluid, hinting at the early neurodevelopmental and altered immune responses in ASD (Pardo et al., 2005; Zimmerman et al., 2005; Skogstrand et al., 2019). Neuroinflammation and oxidative stress of ASD trigger cell connection breakdown and cytoskeletal recombination of ECs, damaging the BBB (Kumar et al., 2009; Daneman and Prat, 2015). Under stressful conditions, the hypothalamus secretes corticotropin-releasing hormone (CRH) and neurotensin (NT) to stimulate the release of VEGF from mast cells in the brain, leading to the increase of BBB permeability, resulting in focal encephalitis that may be associated with an increased incidence of ASD (Theoharides, 2013). Some pathological states associated with the development of ASD for example, gastrointestinal abnormalities and perinatal infections may also lead to abnormalities in the cerebrovascular and BBB. A hypothesis based on the gut-brain axis suggested that antigen and immune complex impairment of the gut barrier and BBB may act as part of subsequent inflammatory activation and neurological disease (Fiorentino et al., 2016; Margolis et al., 2021; Socała et al., 2021). Bile acids and lipopolysaccharide (LPS) produced by bacteria can induce the release of cytokines to affect BBB permeability (Quinn et al., 2014; Goyal et al., 2021). Interestingly, bacterial fermentation products short-chain fatty acids (SCFAs) affect the maturation of CNS microglia as signaling molecules within the nervous system (Erny et al., 2015). Moreover, maternal viral infection can be directly or indirectly involved in the mechanism of BBB defects. Dengue virus disrupts the glycocalyx of endodermal (Puerta-Guardo et al., 2016), and Zika virus and Herpes Simplex Virus release cytokine TNFα, both of which can downregulate the tight junction protein expression and alter BBB integrity (Chiu et al., 2020; He et al., 2020). Inflammatory cytokines (e.g., IL-6 and TNF) released by maternal viral infection during pregnancy can cross the placenta (Meyer et al., 2009) and act as a neuroimmune “switch” for ASD, beginning to activate the inflammatory cascade of microglia, affecting brain and BBB development with behavioral abnormalities (Brudnak, 2001; Zawadzka et al., 2021). Collectively, abnormal neuroinflammation and oxidative stress in the brain are consistently observed as co-morbid conditions in ASD, closely associated with BBB dysfunction, and collectively contribute to the pathophysiology of ASD. Abnormal immune activation in the CNS is involved to some extent in BBB damage, while systemic inflammation may impact normal brain development due to BBB dysfunction in individuals with ASD.

### 3.3. Altered neurovascular unit in autism spectrum disorders

#### 3.3.1. Altered hemodynamic responses in autism spectrum disorders

Although localized to CNS endothelial cells, its function also relies on the collaboration of other cell types, including pericytes and astrocytes. These cells together with neurons and smooth muscle cells form a multicellular system, termed NVU, to regulate CBF as needed to coordinate brain function (Langen et al., 2019). Individuals with ASD have been observed to exhibit smaller hemodynamic responses in specific brain regions, such as the dorsolateral prefrontal cortex, bilateral ventrolateral prefrontal cortex, and anterior temporal cortex, during various tasks (Kawakubo et al., 2009; Hosokawa et al., 2015; Uratani et al., 2019; Ohtani et al., 2021). This change was reported in children with ASD during color-word task, auditory tasks, and self-face recognition using near-infrared spectroscopy (NIRS) measurements (Kita et al., 2011; Funabiki et al., 2012; Uratani et al., 2019). In the letter fluency task, measurement of relative hemoglobin concentrations in the prefrontal cortex revealed no significant change in the child group with ASD, but instead decreased in the adult group (Kawakubo et al., 2009). However, there are studies showing different result (Feczko et al., 2012; Xiao et al., 2012). Hemodynamics itself is heterogeneous across brain regions and subjects and is influenced by both neural activity and non-neural factors (Reynell and Harris, 2013; Yan et al., 2018). Therefore, it is uncertain whether hemodynamic changes in these studies directly reflect abnormalities in NVU. Notably, a recent study reliably confirmed the presence of neurovascular uncoupling in 16*p*11.2^*df*⁣/ +^ mouse model of ASD. The experiment increased neuronal activation by stimulating whiskers, but induced a weaker vascular response in mutant young adult male mice (Ouellette et al., 2020). After excluding abnormalities in vascular smooth muscle cell function, it was postulated that these neurovascular impairments may be a result of defective vasodilation caused by endothelial deficit (Ouellette et al., 2020). Overall, these studies suggest that hemodynamic abnormalities are found in patients with ASD, affecting the supply of oxygen and nutrients to some brain regions, possibly due to NVU defect.

#### 3.3.2. Altered elements of neurovascular unit in autism spectrum disorders

The contribution of the composition of NVU, including ECs, pericytes, astrocytes, and microglia, to proper brain development and function, is being increasingly elucidated (Carrier et al., 2020). Various elements of the NVU have been found to be disrupted in recent years in ASD. The potential impairment of vascular endothelial in ASD has been mentioned in individual research, characterized by downregulation of angiogenic genes in ECs and endothelium-dependent vasoconstriction abnormalities (Ouellette et al., 2020). In addition, it has been reported that oxidative stress in ASD increases the formation of F2-isoprenaline leading to platelet and vascular endothelial activation (Yao et al., 2006). The interaction of ECs and nerve cells ensures proper brain function (Segarra et al., 2018). Although VEGF-A is a pro-angiogenic substance secreted primarily by neuronal cells, it is also expressed in ECs to regulate the migration and localization of neurons during early development, as well as the interneuron spatial association to vessels. Vascular-specific knockout of VEGF-A contributed to impaired angiogenesis, defects in cortical cytoarchitecture, and axonal tracts in the telencephalon (Li et al., 2013). Similarly, GABA is also partly secreted by the vascular system and influences the migration of cortical neurons during embryonic development (Li et al., 2018). When the secretion of GABA in vascular ECs is deficient, the interruption of GABA signal in ECs and neurons leads to impaired forebrain development, affecting normal angiogenesis, neurogenesis, projection neurons, and the migration of GABAergic neurons, resulting in autistic behaviors (Li et al., 2018; Choi and Vasudevan, 2019). Considering the importance of the vascular endothelium, its contribution in ASD is gaining more and more attention. Moreover, pericytes are embedded in the basement membrane outside the endothelium of small vessels, including capillaries (Winkler et al., 2011). Pericytes have been reported to be involved in regulating NVU and BBB integrity, angiogenesis, regulation of CBF, and neuroinflammation (Zehendner et al., 2013; Sweeney et al., 2016). A 2016 immunocytochemical study of post-mortem brain sections from ASD identified elevated expression of nestin in pericytes in specific brain region, indirectly indicating continued angiogenesis after normal development in ASD patients (Azmitia et al., 2016). Mercier et al. (2011) also found vascular alterations and associated extracellular matrix changes in the lateral ventricular neurogenic area of adult BTBR T+ tf/J mice, showing arterial enlargement and choroid plexus atrophy. Furthermore, astrocytes are the most abundant cells in the CNS. In addition to the traditional function of providing support to neurons, differentiated astrocytes can release signaling molecules such as Ang-1 and TGF-B to promote BBB maturation (Lee et al., 2003; Daneman et al., 2009). The characteristic structure of the perivascular astrocyte foot processes, orthogonal arrays of particles (OAPs), plays an important role in the homeostasis of the glial-endothelial interface (Wolburg et al., 2011). Two markers of abnormal glial-neuronal communication, aquaporin 4 (an important component of OAP) and connexin 43, both were found to be altered in ASD subjects, possibly associated with increased BBB permeability and neuroinflammation (Fatemi et al., 2008). Besides, S100B and glial fibrillary acidic protein (GFAP) values expressed by astrocytes in the serum of children with ASD are higher than those of normal controls and are expected to be an indicator of the severity of neurological damage (Al-Ayadhi and Mostafa, 2012; Esnafoglu et al., 2017). In addition, microglia is a special cell type in NVU, which are resident macrophages of brain tissue that develop from hematopoietic progenitor cells in yolk sac before the BBB closure (Nayak et al., 2014; Colonna and Butovsky, 2017). Microglia also can affect neuronal proliferation, migration, and programmed death, promotes the construction of neural circuits and myelination, now are also thought to have the ability to promote vascular germination and branching (Fantin et al., 2010; Rymo et al., 2011; Ganguli and Chavali, 2021). Recent studies suggest that microglia in ASD patients are more active, and show characteristic amoeboid patterns that can present antigens, release more cytokines, and recruit white blood cells in blood vessels (Zlokovic, 2008; Morgan et al., 2010; Suzuki et al., 2013). Some scholars attribute the stereotypic behavior and social dysfunction of ASD patients to the effect of microglial activation and leukocyte infiltration on neural development, leading to neurological dysfunction (Zhan et al., 2014). Platelets are recruited under the damaged endodermis during neuroinflammation, releasing inflammatory mediators and recruiting other inflammatory cells, exacerbating the inflammatory process (Deppermann, 2018). The CNS has long been regarded as an immune privilege site due to the presence of BBB, but the brain cannot be separated from the peripheral immune system (Zengeler and Lukens, 2021). The effects of neuroimmune on CNS development are multifaceted. Normal immune cells are necessary for CNS development, such as astrocytes can participate in synapse formation and regulation (Volterra and Meldolesi, 2005). However, abnormal neuroinflammation in the ASD brain can cause extensive damage to the constituent cells of the NVU. Therefore, it is reasonable to hypothesize that impairments in cerebrovascular features and immune activation interactions play a significant role in the pathophysiology of ASD.

## 4. Discussion and prospect

Indeed, these cerebrovascular characteristics mentioned earlier are not independent in ASD. Proper cerebral blood supply relies on both normal cerebrovascular network and regulation of neurovascular coupling. Disruptions in either of these factors can impair brain maturation by compromising the oxygen and blood supply necessary for proper brain function. Furthermore, systemic inflammation and immune activation have long been associated with the pathogenesis of ASD as co-morbid conditions. One potential mechanism is inflammation, derived from environmental factors (e.g., gut-brain axis dysbiosis, perinatal maternal infections, vaccines) and genetic susceptibility, as a source of cerebrovascular susceptibility (Theoharides, 2013; Zengeler and Lukens, 2021). Indeed, neuroinflammation could damage the component cells of NVC. Defective NVC cells are unable to maintain the integrity of the BBB, resulting in BBB leakage and inflammatory mediators and cells from the periphery easily entering and damaging the brain. This mechanism has been demonstrated in other psychiatric disorders, such as schizophrenia and Alzheimer’s disease (Carrier et al., 2020; Korte et al., 2020).

The vascular and nervous systems construct a highly specialized system separately, but share overlapping mechanisms during embryonic development. It is plausible that abnormalities in vascular development could adversely affect associated neurodevelopmental processes. Regrettably, these studies that have explored the neurovascular interactions of ASD are largely non-developmental, providing only a snapshot at a single point in time (Azmitia et al., 2016; Fiorentino et al., 2016). For example, studies on cerebrovascular alterations in patients with ASD have been conducted using post-mortem brains (Azmitia et al., 2016; Fiorentino et al., 2016), making it challenging to determine the timing of these defects. Although these studies have identified cerebrovascular vasculature as an important component of ASD, it is unclear whether changes in vascular structure and function are a cause or a consequence of neuropathy in ASD. More research is needed to further explore the pivotal role of cerebrovascular defects in the neuropathology of ASD. Additionally, the limited sample sizes in these studies warrant caution, considering the complex pathophysiology and heterogeneous phenotype of ASD.

However, recent studies have provided evidence of cerebrovascular alterations in individuals with ASD (Azmitia et al., 2016; Fiorentino et al., 2016; Bjørklund et al., 2018). Hypoperfusion and neuroinflammation caused by cerebrovascular alterations may emerge as a new potential pathogenesis of ASD. Thus, conceptually, treatments that improve intracerebral hypoperfusion or restore defective BBB and NVU may show beneficial effects in patients with ASD. In fact, several studies based on the repair of cerebrovascular features have made many new advances. As previously described, perfusion abnormalities in specific cortical regions are associated with key features of ASD (Ohnishi et al., 2000; Bjørklund et al., 2018). Improve hypoperfusion through vasodilatation or pro-angiogenesis may be useful. Considering that the VEGF pathway plays an important role in neurovascular communication, more and more studies have focused on this pathway as a potential therapeutic target of neurodevelopmental disorders and neurodegenerative diseases. In mice experiment, VEGF-C injections were found to dilate blood vessel diameter, restore cerebrospinal fluid flow, and ultimately improve cognitive test results (Da Mesquita et al., 2018). Besides, transplantation of VEGF-A-producing cells has been shown to promote the repair of brain damage caused by ischemia in neonatal rats (Yao et al., 2016). Other studies indicate that intrathecal transplantation of autologous bone marrow mononuclear cells (BMMNCs) in 32 patients with autism has shown significant clinical effects by promoting angiogenesis to improve perfusion and balance inflammation through immunomodulation (Sharma et al., 2013). Notably, the persistent proliferation of blood vessels in the brain of ASD patients after normal development may be related to the disorder of brain functional connectivity and the increased incidence of epilepsy. Anti-VEGF therapies may reduce abnormal neuronal activity in ASD patients by inhibiting vascular plasticity (Greenberg and Jin, 2005; Shibuya, 2009; Azmitia et al., 2016). However, given the high heterogeneity of pathophysiological conditions in individuals with ASD, further studies are warranted to evaluate the feasibility and effectiveness of pro/anti-angiogenic therapies in this population.

In addition, restoring the integrity of the BBB may also benefit patients with ASD by re-establishing vascular function and providing neuroprotection. Study has demonstrated that once-daily administration of P7C3-A20, a compound that stabilizes cellular energy levels, is beneficial to the recovery of BBB endothelial injury, neuroinflammation reduction, and cognitive recovery after traumatic brain injury (TBI) (Vázquez-Rosa et al., 2020). Alvarez et al. (2011) claimed that shh secreted by astrocytes binds to receptors in BBB ECs, has a BBB protective effect. Tumor necrosis-alpha (TNF-α) and interferon-gamma (IFN-γ) -treated astrocytes increased Shh production, to promote BBB repair and balance inflammation induced during BBB injury (Alvarez et al., 2011). This study provides new avenues for designing treatments to repair BBB. Balancing neuroinflammation in the brain is also beneficial for defective BBB and NVU. For example, pioglitazone is a non-steroidal anti-inflammatory drug (NSAID) was found can improve ASD symptoms by reducing microglial activation and subsequent inflammation in children with ASD (Emanuele et al., 2007). Immunoregulatory therapy may be an effective option as a personalized treatment for patients with a distinct inflammatory phenotype (Pape et al., 2019).

Moreover, interventions targeting ASD risk genes associated with neurovascular development, are also expected to provide potential therapeutic benefits in the management of ASD symptoms (Baranova et al., 2021). The Wnt/Shh pathway is critical for controlling neural progenitor cell differentiation, neuronal migration, synaptogenesis, NVU development, and BBB formation, the disruption of the pathway may lead to neurodevelopmental disorders including ASD (Gozal et al., 2021). Therapeutic interventions based on components of the Wnt/Shh pathway are currently being tested or used for the treatment of various neurological diseases (Baranova et al., 2021; Rahi and Mehan, 2022).

## 5. Conclusion

This review focuses on neurovascular communication during embryonic development and recent research advancements regarding cerebrovascular alterations in ASD, aiming to deepen our understanding of ASD pathogenesis. We also discuss the possibility of these findings as a mechanistic basis for vascular-based therapy for ASD. As we remarked above, these treatment attempts have yielded some positive results, which further confirm the presence of cerebrovascular defects in ASD individuals. Although these current treatments targeting cerebrovascular structure and function do not provide a cure for autism, it is certain that they have great potential to control disease progression and improve quality of life. The investigation of ASD has increasingly emphasized the role of the cerebrovascular component. These studies provide additional evidence for exploring the mechanisms underlying ASD and demonstrate that ASD can be considered as a neurovascular disease. The cerebral vasculature is crucial for normal brain function, and emerging evidence has linked it to various neurological disorders. It is curious that similar vascular abnormalities are found in neurological diseases with different pathogenesis and clinical features. Cerebrovascular defects make the brain more vulnerable, and it is reasonable to assume that the vascular plays a more important role than previously thought. However, much of the evidence supporting the vascular basis of neurological disease needs to be explored in additional causal studies, to determine the extent to which cerebrovascular alterations are involved in pathogenesis or whether these changes do as a result of pathophysiological changes.

## Author contributions

YW: writing-original draft. YW, SY, and ML: writing-review and editing. All authors have read and agreed to the published version of the manuscript.
