# Corrigendum: Neurovascular crosstalk and cerebrovascular alterations: an underestimated therapeutic target in autism spectrum disorders

**DOI:** 10.3389/fncel.2024.1435981

**Published:** 2024-07-05

**Authors:** Yiran Wang, Shunyu Yu, Mengqian Li

**Affiliations:** ^1^Queen Mary School, Jiangxi Medical College, Nanchang University, Nanchang, China; ^2^Department of Psychosomatic Medicine, The First Affiliated Hospital of Nanchang University, Nanchang, Jiangxi, China

**Keywords:** autism spectrum disorder (ASD), cerebrovascular, blood-brain barrier, neurovascular unit, neurovascular crosstalk

In the published article, there was an error in section **3.1 Impaired cerebral blood supply in autism spectrum disorders**, subsection *3.1.1 Altered cerebral blood flow in autism spectrum disorders*, in the heading title, paragraph 1 and paragraph 2 in which insufficient paraphrasing resulted in inaccurate expression. The corrected text appears below:

“3.1.1. Insights into cerebral blood flow changes in autism spectrum disorders”

“Common imaging techniques such as Functional magnetic resonance imaging (fMRI), Arterial Spin Labeling (ASL), single-photon emission computed tomography (SPECT), Positron Emission Tomography/Computerized Tomography (PET/CT), and an emerging technique Diffuse correlation spectroscopy (DCS) (Lin et al., 2023) have been used to detect regional cerebral blood flow (rCBF) and can significantly enhance our understanding of the contribution of brain vasculature to ASD.”

“A case-control study showed that significantly higher rCBF values were prevalent in specific regions in patients with ASD, and the higher the rCBF value, the more severe the socialization deficit. This may be due to alterations in metabolism and axonal function that reduce the role of nerves in cognitive and social functioning, which provokes a compensatory response from glial cells, that results in rCBF increase (Peterson et al., 2019).”

The authors apologize for this error and state that this does not change the scientific conclusions of the article in any way. The original article has been updated.
